# From Biosensors to Robotics: Pioneering Advances in Breast Cancer Management

**DOI:** 10.3390/s24186149

**Published:** 2024-09-23

**Authors:** Mohd. Rahil Hasan, Mohd Mughees, Shifa Shaikh, Furqan Choudhary, Anam Nizam, Amber Rizwan, Onaiza Ansari, Yusra Iqbal, Roberto Pilloton, Saima Wajid, Jagriti Narang

**Affiliations:** 1 Department of Biotechnology, Jamia Hamdard, New Delhi 110062, India; rahilhasan789@gmail.com (M.R.H.); mmughees@mdanderson.org (M.M.); shaikhashifashaikh1599@gmail.com (S.S.); furqaan013@gmail.com (F.C.); nizamanam@yahoo.in (A.N.); amberjh399@gmail.com (A.R.); ozansari8@gmail.com (O.A.); yusraiqbal1996@gmail.com (Y.I.); 2 CNR-IC, Area della Ricerca di RM1, Via Salaria km 29.3, Monterotondo, I-00015 Rome, Italy

**Keywords:** breast cancer, biosensor, therapy, surgery, therasurgynostic

## Abstract

Breast cancer stands as the most prevalent form of cancer amongst females, constituting more than one-third of all cancer cases affecting women. It causes aberrant cell development, which can assault or spread to other sections of the body, perhaps leading to the patient’s death. Based on research findings, timely detection can diminish the likelihood of mortality and enhance the quality of healthcare provided for the illness. However, current technologies can only identify cancer at an advanced stage. Consequently, there is a substantial demand for rapid and productive approaches to detecting breast cancer. Researchers are actively pursuing precise and timely methods for the diagnosis of breast cancer, aiming to achieve enhanced accuracy and early detection. Biosensor technology can allow for the speedy and accurate diagnosis of cancer-related cells, as well as a more sensitive and specialized technique for generating them. Additionally, numerous treatments for breast cancer are depicted such as herbal therapy, nanomaterial-based drug delivery, miRNA targeting, CRISPR technology, immunotherapy, and precision medicine. Early detection and efficient therapy are necessary to manage such a severe illness properly.

## 1. Introduction

Cancer is recognized to undergo a complex step-by-step oncogenesis mechanism that involves various cellular physiological processes like cell signaling and programmed cell death, transforming it into a highly enigmatic and intricate ailment. Although it starts as a localized illness, cancer is lethal because it can easily extend to other body parts. There are many different varieties of cancer, encompassing cervical, ovarian, breast, and pancreatic cancer, and many more. Cancer of the breast holds a prominent position as one of the globally recognized malignancies [1,2]. BC (breast cancer) is a metastatic disease that can also affect bone, the liver, lungs, and brain, which explains why it is incurable. Regarding its frequency of occurrence and mortality rates, breast cancer stands as the primary malignancy affecting women, followed by colorectal and lung cancer. Approximately twenty percent of worldwide cancer cases and fourteen percent of global cancer-associated fatalities are attributed to the United States. In India, breast cancer represents nearly 5–8% of the total cases of cancer, and a noteworthy proportion of breast cancer patients, specifically 20%, exhibit a potential for survival up to five years without receiving therapeutic interventions [3,4]. Breast cancer is also considered a heterogeneous illness, since it represents a variety of phenotypically distinct tumors [5]. BC (breast cancer) affects not just women, but also males. Male breast cancer is uncommon; however, it shares several epidemiologic features with female breast cancer. Although data is limited at this time, men with Klinefelter’s syndrome, gynecomastia, and testicular disease and dysfunction have an elevated risk of breast cancer [6]. More than 3500 years ago, ancient Egyptians were the first to notice breast cancer [7]. During the fifth to fourth centuries BC, Hippocrates, the father of Western medicine, considered breast cancer to be a humoral illness. He documented the first actual case of breast cancer in history, which occurred in an Abdera woman. She was diagnosed with cancer after presenting with chest wall growth and bleeding from the nipple. She died after the bleeding stopped. Hippocrates also mentioned the presence of hard lumps in the breasts, which he thought may have developed into concealed malignancies, which were recognized as breast cancer later on [8]. The pink ribbon, as a breast cancer logo, sparked a movement fighting the disease in the 1990s [9]. In addition, World Breast Cancer Awareness Day is observed in October to promote awareness and raise finances for breast cancer sufferers [10]. Despite significant development in the field of molecular oncology, no appropriate and effective therapies for this illness are now available. Traditional therapies are available, but while they considerably postpone tumor growth, recurrence is unavoidable, resulting in high fatality rates [11]. The timely identification of breast cancer stands as a highly efficacious approach to preventing the onset of the disease. As a consequence of prompt identification, certain industrialized nations exhibit a relative survival rate of 80% over the five years among breast cancer patients [12]. So, researchers try their hardest to make breast cancer more curable by developing different approaches in various fields such as diagnosis. “Biosensor tools” are a more prominent way to diagnose breast cancer at an early stage [13]. In therapy, “Immunotherapy” is effectively recommended for breast cancer treatment, and surgery is referred to as the last hope to cure the disease by removing cancer from the body. “Robotic surgery” is immensely popular nowadays, as it helps to remove the cancer from the patient’s body with minimally invasive perfections [14,15].

**Figure 1:**
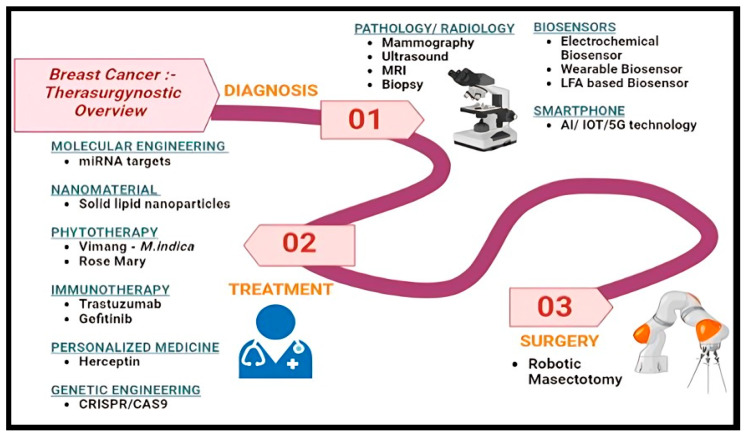
Diagrammatic representation of effective “Therasurgynostic for Breast Cancer Management”, comprising smart diagnostics, effective therapy, and advanced surgery, which incorporate various approaches from biosensors to robotics to make a more effective therapy.

Therefore, in this review, we have explored various smart detection, innovative therapies, and robotic surgery techniques for breast cancer management, which leads to the new term called “Therasurgynostic”, which encompasses diagnostic and therapeutic research followed by advanced surgery procedures under one title (Figure 1). Therasurgynostic has the potential to greatly enhance BC care, contributing to the worldwide effort to control and ultimately eradicate this dreadful disease.

## 2. Diagnosis Methods for the Detection of Breast Cancer 

The diagnostic stage holds significant prominence in the comprehensive management of breast cancer. Traditional techniques for detecting breast cancer include mammography, MRI, ultrasonography, and biopsy. 

(a) Mammography is still the only imaging modality that is recommended for regular breast cancer screening in people of all ages. Screening mammography must be conducted regularly to be effective in lowering breast cancer mortality, as demonstrated by multiple randomized controlled studies [16]. (b) Ultrasonography, a typical screening method, uses the reflection of sonic waves from breast tissue to detect malignancies. For females at elevated risk for BC, pregnant women, or those who are incapable of undertaking mammographic screening, breast ultrasound has been promoted as an additional modality to mammography. It augments imaging sensitivity while simultaneously resulting in reduced specificity and increased rates of biopsy as a trade-off. (c) MRI: By integrating a powerful magnetic field with radiofrequency (RF) signals, magnetic resonance imaging (MRI) produces images from multiple cross-sectional perspectives. Additionally, the quality of the MRI picture can be improved by the addition of a contrast agent. Breast MRI has been recommended for individuals deemed at greater danger of emerging breast cancer. Compared to mammography and ultrasound, MRI exhibits greater sensitivity but lower specificity in the detection of minuscule tumors among those at elevated risk of BC [17]. (d) Biopsy: Tissue biopsy is an invasive process that neither captures the entire genetic landscape of breast tumors nor is useful for assessing therapy response. Another sort of biopsy is a liquid biopsy. This less-invasive molecular technique serves as an advanced method for monitoring advanced cancer, based on analysis of genetic elements extracted from tumor cells and put into the environment [18].

The aforementioned section on diagnosis discusses the different methods to detect breast cancer, but it meets several limitations. Table 1 summarizes various advantages and limitations of diagnostic methods. Thus, the need arises for an early detection tool that is swift, precise, sensitive, user-friendly, and non-invasive, i.e., biosensor, which is discussed in the subsequent section.

### Biosensor: Innovative Tool for the Effective and Early Diagnosis of Breast Cancer

Clark and Lyons developed the first biosensor to measure the glucose amount in biospecimens in the year 1962. A biosensor is an object of bio-analytical technology made up of three components: a signal transducer, a recognition component, and a signal amplifier which transfers and displays the results. Previously, biosensors for precise and sensitive BC diagnosis were created [1], like electrochemical biosensors, wearable biosensors, LFA-based biosensors, and smartphone application-based biosensors (Table 2).

(a) Electrochemical biosensor designed for BC diagnosis

**Figure 2:**
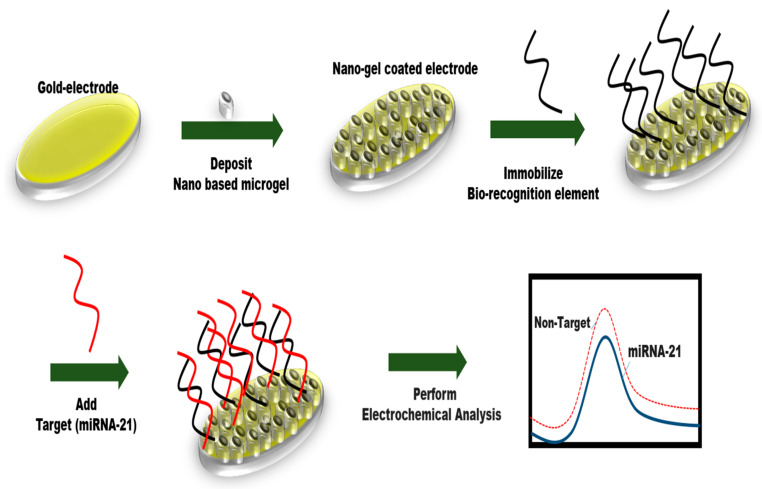
The electrochemical analysis of the breast cancer biomarker, miRNA-21, consists of many processes, including nanogel deposition on the electrode surface, immobilization of the probe to capture the selected target, addition of the breast cancer biomarker, and electrochemical analysis for miRNA-21 detection.

These biosensors have an electrochemical transducer and use various electrochemical procedures to detect current via chemical solutions (electrolytes). These measures are classified into three types: Impedimetric (change in impedance), Amperometric (change in current), and Voltammetric (change in potential, for example, CV/SWV/DPV, and so on). Many writers have used electrochemical approaches to identify breast cancer biomarkers [24]. Various biomarkers were accessible. However, miRNA is a common biomarker for breast cancer and is regarded as a superior indicator due to its potential as an initial stage predictor for the detection of BC [25]. Cardoso et al. [26] designed an electrochemical biosensor specifically to detect miRNA-155 (a small non-coding microRNA), showing a great limit of detection, e.g., 84.3 fM with no cross-reactivity. In this work, firstly, they immobilized the probe (single-stranded-DNA) on the surface of the sensor and then added the target to it. Further electrochemical analyses were performed with the help of methylene blue, which is used as a redox indicator. Electrochemical analysis of the developed sensor (probe–target hybrid) was successfully validated by three approaches: (a) CV, i.e., cyclic voltammetry, (b) EIS, i.e., impedance spectroscopy, (c) DPV, i.e., differential pulse voltammetry. The validation of the methylene blue response was additionally confirmed through the implementation of cyclic voltammetry (CV) and chronocoulometry techniques. These analyses unveiled a linear correlation between the redox-peak currents (i.e., Ip) and the scan -ate (i.e., ν), showing that the transferring of electrons occurring amid the methylene blue and the surface of the electrode was facilitated through the miRNA/DNA π-stacked duplex. The determination of surface coverage value, i.e., Γ, demonstrated an elevated quantity of electrolytes upon the altered electrode surface following the hybridization result. This finding elucidated the occurrence of monolayer adsorption of electrolytes on the modified electrode. Furthermore, the estimation of electron transfer rate constants (ks) for methylene blue was performed. Consequently, this approach holds significant promise for achieving a highly specific and label-free diagnosis of miRNA. Recently, Hou et al. [27] fabricated an electrochemical biosensor-based n aptamer for the identification of miRNA-21 (serum) and showed LOD, i.e., 1.35 aM. In this study, the researchers fabricated a nanocomposite material consisting of microgel particles incorporating gold nanoparticles (GNPs), which were enveloped within a polymerization mixture comprising acrylic acid and N-isopropyl acrylamide. Subsequently, the synthesized nanocomposites were deposited onto the sensor surface. Immobilization of biological recognition element (RNA) on the surface of the nanoparticles was obtained by the covalent binding procedure. At last target (miRNA-21) was detected by adding it to the aptamer functionalized electrode and confirming the detection of the target with the help of DPV. The outcomes were also successfully checked in two breast cancer patients. The aptasensors that were developed exhibited remarkable capability in detecting breast cancer serum biomarkers at an initial phase in real samples without the need for sample purification and preparation (Figure 2).

(b) Wearable biosensor for breast cancer diagnosis

A multitude of physical biosensors have been developed thus far, including wearable or lab-on-body devices that establish a direct interface with the skin to monitor vital signs, movement, step count, calorie expenditure, and heart rate. Recent advancements and innovations in non-invasive biomarker screening encompass the assessment of metabolites, microbes, and hormones, demonstrating that biosensors may be incorporated into wearable systems [28]. Breast cancer can be properly diagnosed via wearable biosensors, which include jewelry-based wearable biosensors, glove-based wearable biosensors, textile-based wearable biosensors, and many more. Breast cancer diagnosis falls under the category of textile-based wearable biosensors. Currently, the application of “smart textiles”, also known as “intelligent” or “wearable technology” through various bases, is rapidly developing and has surfaced in a variety of textile domains like apparel, household, and technical textiles. The fundamental base of smart textiles lies in the all-in-one incorporation of micro-electro-mechanical systems (MEMS) in diverse embodiments, such as microchips, software, sensors, and interconnections, within fabric materials [29]. The “smart bra” is a unique wearable biosensor of breast cancer, and many reports have been proven by famous researchers towards breast cancer smart diagnosis.

**Figure 3:**
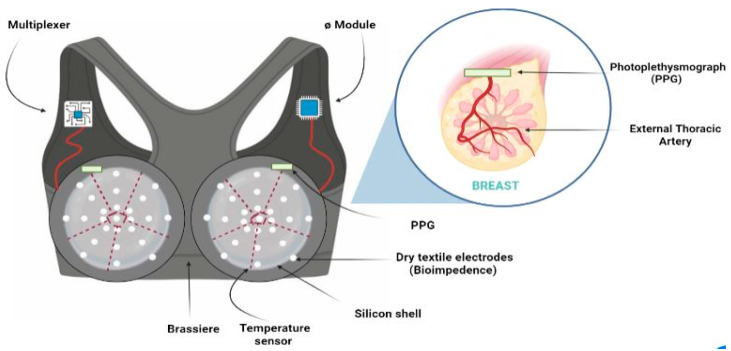
Diagrammatic representation of a smart-bra-based wearable biosensor for breast cancer diagnosis, comprising the thermal sensor connected with the target site.

A smart bra for early detection of breast cancer will be available soon in the market. The ultrasonic technology used in the gadget emits no radiation. According to the innovator, a Nigerian-developed smart bra device can detect breast cancer at an initial stage, potentially reducing the need for African women to travel long distances in search of screening facilities. Kemisola Bolarinwa, a robotics engineer who created this smart bra, thinks it will help to overcome the hurdles to early identification of the disease [30]. Other than Nigeria, England also initiated the concept of a smart bra that can recognize early tissue changes in the breasts, like inflammation or changes in the flow of blood, that take place before tumor recognition. This smart bra has been developed by Elias Siores, Ph.D., who is the director of the department of the Centre for Materials Research and Innovation at the University of Bolton. To detect these alterations at the target spot, the proposed sensor makes use of a network of microwave antennas [31]. In another literature, Julian Rios Cantu (an 18-year-old girl), did more than just dream it. She recruited a team and developed a breast cancer sensor prototype called EVA. Her investigation was motivated by her mother’s fight with cancer. She is now the CEO of Higia Technologies, a Mexican-based biosensors firm dedicated to the initial diagnosis of BC (breast cancer). The sensor resembles any other bra. It does, however, have 200 tiny tactile biosensors that map the surface of each breast. It detects variations in texture, color, and temperature [32]. Breast cancer surgeon Dr. Jay Harness (St. Joseph Hospital in California’s Center for Cancer Prevention and Treatment) established a new bra that appears to operate by attempting to detect temperature changes in the skin as a result of increased blood flow, which is potentially linked to the existence of breast cancer [33]. The ongoing development involves a schematic prototype of a bra, wherein all wires joining the electrodes, temperature sensors, and photoplethysmography (PPG) with multiplexers are encased within a silicone shell positioned among the double fabric layer, thereby creating the enclosure [34] (Figure 3). There are not enough reports available on wearable breast cancer biosensors; only a few researchers were reported, such as Manhood et al. [35], who demonstrated a wearable antenna-based bra sensor for the diagnosis of BC. In their study, they employed a miniaturized antenna (MIMO) that performed two functions for both breast cancer imaging and WBAN. WBAN is a system that helps to detect human body conditions and is considered a lightweight, highly powerful wearable sensor, which is easily applied as off-body/in-body/on-body. The performance of the antenna was assessed under both on-body (breast) and free-space circumstances utilizing near-field microwave imaging. The obtained outcomes, which encompassed high fidelity, low specific absorption rate (SAR), and precise tumor localization, suggest that the MIMO antenna holds promise as a viable option for breast cancer imaging. In another study, Fadillah et al. [36] built a wearable gadget for the determination of breast cancer situations. In this project, they attached thermal sensors to brassiere (bra) fabric, which monitored temperature variations in the breasts. The results show that, in comparison to the surrounding areas, the thermal sensors placed on every quadrant with a tumor read higher temperatures. It has an exposed thermistor with improved sensitivity and reaction rate, allowing it to monitor skin temperature. The sensor detects thermal radiation emitted from the skin’s surface. Nevertheless, meticulous positioning and design of the sensors on the bra material are crucial to ensure optimal accuracy. To verify the reliability of the newly developed device, its outcomes were validated by comparing them with those obtained from an established infrared thermal imaging camera.

(c) Lateral flow assay kit-based breast cancer-biosensor

**Figure 4:**
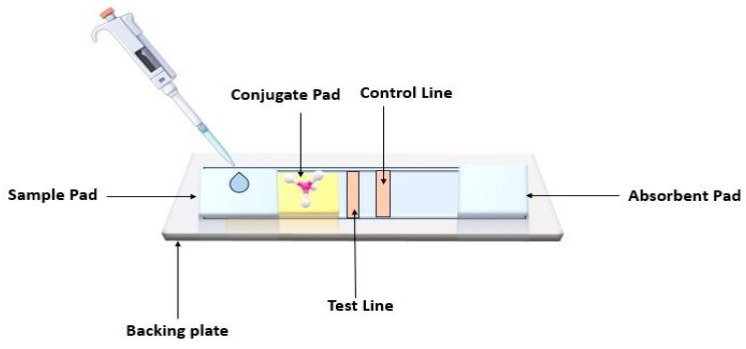
LFA kit for breast cancer detection first adds the sample to the sample pad and moves it to the conjugate pad, where the sample forms a complex with gold nanoparticle-conjugated bio-recognition elements. The complex then flows lateral to the nitrocellulose membrane, and the sample is tested, yielding positive or negative results.

This paper gadget that detects glucose in the urine was introduced in 1956 [37]. The pregnancy kit is a prominent use of LFA [38]. Paper-based lateral flow assay (LFA) kits are widely recognized as point-of-care (POC) devices and have recently been found to be a highly effective approach for the qualitative, semiquantitative, and quantitative diagnoses of several targets, particularly biomarkers. The fundamental procedure for conducting this test involves depositing the sample onto the designated sample pad, where it undergoes characterization or analysis. To optimize detection, the sample is transferred to a conjugate pad where it undergoes multiple interactions, before being subsequently transported to the membrane. The membrane is composed of two main lines, namely the control and test line. Subsequently, the analyte reaches the absorbent pad, designed with adequate bed volume to ensure complete sample flow. After the incubation period, the presence of a solo line on the platform indicates the positive or negative outcome of the assay (Figure 4) [39]. 

As we know, miRNA is one potentially useful biomarker of breast cancer, and it comprises various types of miRNas. Zheng and their co-workers [40] built an LFA that depends upon nucleic acid to detect different microRNAs (21/155/210) and showed a great limit of detection, i.e., 0.073/0.061/0.085 nM, respectively. In this study, the researchers employed the sandwich principle of nucleic acid hybridization reactions to generate complexes consisting of GNPs attached to ssDNA–microRNA–ssDNA structures. These complexes were subsequently photographed and observed on the test zone of the strip. Through the strategic design of three distinct test zones on the strip, the instantaneous determination of microRNAs (21/155/210) was accomplished. This was accomplished by utilizing a model that involved adding and measuring, incorporating GNPs as a visual indicator. The efficacy of this method was tested by analyzing microRNAs in spiked serum samples, yielding satisfactory results. From our perspective, this test represents a highly useful instrument for clinical and medical diagnostic applications, especially in resource-constrained situations. Recently, Dyan B [41] developed a nucleic acid-based lateral flow assay (LFA) specifically for breast cancer diagnosis. This study investigated three well-established target genes associated with breast cancer: the tumor suppressor and transcription factor (p53), the phosphatase and tensin homolog (PTEN), and the breast cancer gene (BRCA1). This assay utilized citrate-capped GNPs as a coloring agent and exhibited an impressive detection limit, namely 0.06 ng/mL for p53 and 0.125 ng/mL for PTEN. The development of the strip involved the immobilization of dig antibody (anti-dig) on the test line and biotin on the control line of a nitrocellulose membrane. Additionally, gold nanoparticles (GNPs) were synthesized using a chemical method. Subsequently, the synthesized gold nanoparticles (GNPs) were conjugated with streptavidin through the application of biotin–streptavidin chemistry. These conjugated GNPs served as the detection probe in the lateral flow assay (LFA). As evidenced by the formation of a pair of red lines on each of the controls and test lines, confirming the presence of the target biomarker, the prepared kit positively recognized the PCR amplicons of p53 and PTEN. Using gold nanoparticles, a quick nucleic acid-based lateral flow assay (NABLFA) was successfully created, demonstrating a remarkable turnaround of 10 min. Alternative terms for “diagnosis” could be “assessment”, “evaluation”, or “examination”. These words convey the idea of determining or identifying a particular condition or disease. However, the above study validates that the LFA based on nucleic acid is one of the promising candidates for the effective assessment of breast cancer.

(d) Breast cancer-based diagnosis via Smartphone AI/IOT/5G technology/cloud computing

**Figure 5:**
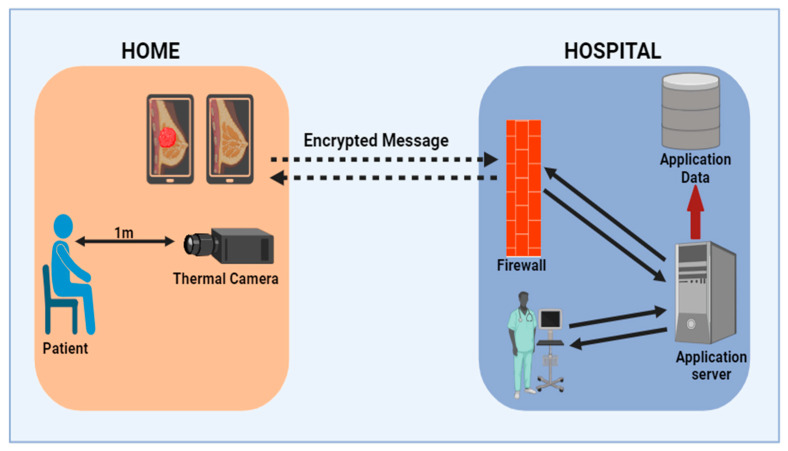
Smart application together with an infrared camera to take photographs and identify breast cancer. To test the app’s functionality, a series of thermal photos were transferred and processed to ensure high-quality images, as they were captured and referred from the mobile device to the cloud server over a variety of distances.

Researchers extensively utilize smartphone applications and artificial intelligence (AI)-based approaches because of their many benefits, which include data analysis, data uploading for cloud-based computing, high-quality picture capturing, multitasking, and on-site testing. Presently, the development of 5G technology is underway, which holds tremendous potential for enhancing the healthcare industry and advancing telemedicine practices. Telemedicine enables patients to receive comprehensive medical assistance without the need to physically go to a hospital. In the coming future, individuals will have the convenience of obtaining affordable testing services from nearby drugstores. Individuals have the option to conduct tests within the comfort of their homes, yielding results within minutes. Subsequently, they can instantaneously and anonymously share their findings with doctors via video chats, significantly reducing the time for each treatment, minimizing the chance of infectious diseases spreading in public settings and allowing doctors to create electronic prescriptions if test findings are abnormal [42]. In more serious cases, doctors may suggest further testing if the outcomes indicate that a dangerous illness, like cancer, is developing early on. There have been several reports of breast cancer diagnosis using smartphone-based innovations. For example, Al Husaini et al. [43] used a smart application together with an infrared camera to take photographs and identify breast cancer. Furthermore, to test the app’s functionality, a series of thermal photos were transferred and processed to ensure high-quality images, as they were captured and referred from the mobile device to the cloud server over a variety of distances. To test the detection accuracy, four effects were performed on the thermal photos: blurring, shaking, tilting, and flipping. The results demonstrate that varied distances and procedures had little effect on thermal picture quality, except for one way that compressed thermal images by 5%, 15%, and 26%. When thermal pictures are compressed by 5%, the findings show that the highest detection accuracy is 1%. Furthermore, the results show that detection accuracy rose by 0.0002% in blurry and shaken photos, whereas diagnosis accuracy declined by approximately 11% in tilted images. The test findings corroborate the proposal’s usefulness, since 100% of breast cancer diagnoses are now correctly predicted. The developed platform concludes that breast thermography employing health apps for devices and cloud computing is an effective technique for initial diagnosis of BC, exclusively in distant locations and with older patients, in addition to giving health information, rapid intervention, and regular follow-up for patients (Figure 5). In another study, Malhotra et al. [44] utilized the IoT, machine learning, and cloud computing technology for the therapy of BC during the COVID-19 outbreak. BC time series forecasting represents an innovative data-driven methodology for the diagnosis of breast cancer. Rather than relying solely on static images of medical records, this approach examines the dynamic patterns in the tumor’s growth rate, with a particular emphasis on its initial stages. The developed model served as a valuable tool in monitoring patients remotely during the COVID-19 pandemic. By utilizing IoT devices, the model provides real-time predictions to both patients and doctors regarding their daily routines. Additionally, machine learning prototypes aid in the classification of BC symptoms, enabling doctors to assess the condition of cancer patients more effectively. Smartphone applications were also integrated with sensors by various researchers such as Zeng et al. [45], who designed a smartphone application reliant on immunoassays for the identification of the BC biomarker HER2. This has led to the successful determination of its optimal range, which spans from 0.01 ng/mL to 10 ng/mL, with a detection limit as low as 3.5 pg/mL. In an alternative study, Low SS et al. [46] have also devised a compact sensor that operates on smartphones to identify microRNA-21 within saliva samples. The biosensing system, which relies on a smartphone as its foundation, comprises an electrochemical sensor integrated with a circuit board for detection purposes. This system is accompanied by a custom-designed Android application, and the smartphone itself is equipped with Bluetooth capabilities. Remarkably, the system demonstrated performance comparable to that of a commercial electrochemical workstation. It successfully detected miR-21 within a concentration range of 1 × 10^−4^ M to 1 × 10^−12^ M, yielding a correlation coefficient (r2) of 0.99. The sensor based on a smartphone platform is designed to achieve a prompt and exceptionally sensitive diagnosis of circulating miRNA biomarkers in bodily fluids. This system is intended for use in inaccessible zones where access to comprehensive medical facilities may be limited.

**Table 2:** Summary of various breast cancer biosensors, comprising advantages, biomarkers, LOD, and their principles.

S. No.	Biosensors with Their Benefits	Principle	Biomarker	LOD	References
1.	Electrochemical (most authenticate and effective biosensor)	Monitoring by using three approaches (cyclic voltammetry/impedance spectroscopy/differential-pulse voltammetry)	miRNA-155	84.3 fM	[26]
		Developed aptasensor based on microgel particles incorporating gold nanoparticles	miRNA-21	1.35 aM	[27]
2.	Wearable (helpful for continuous monitoring of breast cancer biomarker)	A bra monitoring structure based on miniaturized wearable antennae	Breast Cancer	high fidelity and precise tumor localization	[35]
		Development of wearable device attached thermal sensors to brassiere (bra) fabric	BC	monitors temperature variations in the breasts	[36]
3.	LFA (highly affordable and portable biosensor)	Sandwich principle of nucleic acid hybridization based on GNPs	microRNAs (21/155/210)	0.073/0.061/0.085 nM	[40]
		Nucleic acid-based lateral flow assay (LFA) utilizing citrate-capped GNPs as a coloring agent.	p53 and PTEN	0.06 ng/mL and 0.125 ng/mL	[41]
4.	Smartphone (effective in pandemic situation or on-site monitoring)	Self-detection of early breast cancer application with infrared camera and deep learning	Breast Cancer	breast cancer diagnoses were correctly predicted	[43]
		Utilizing the IoT, machine learning, and cloud computing technology for the therapy of BC during the COVID-19 outbreak	Breast Cancer	-	[44]
		Smartphone-based detection followed by photo-electrochemical immunoassay	HER2	3.5 pg/mL	[45]
		The portable system is accompanied by a custom-designed Android application, and the smartphone itself is equipped with Bluetooth capabilities	microRNA-21 in saliva	concentration range of 1 × 10^−4^ M to 1 × 10^−12^ M	[46]

## 3. Therapy to Cure Breast Cancer 

Researchers try their best to investigate effective therapies against breast cancer so that the patient will easily overcome such a painful disease and get their healthy life back. This section encompasses a comprehensive overview of the diverse therapeutic approaches that have been employed for BC treatment (Table 3). 

### 3.1. Traditional Molecular Engineering-Based Therapy 

Molecular engineering has been a longstanding discipline utilized in the treatment of numerous cancer types, like BC. In this field, scientists use different approaches to cure the disease such as plant-based therapy, nanomaterial-based therapy, and miRNA targeting-based therapy.

(a) Plant/Herbal-Based Breast Cancer Therapy

**Figure 6:**
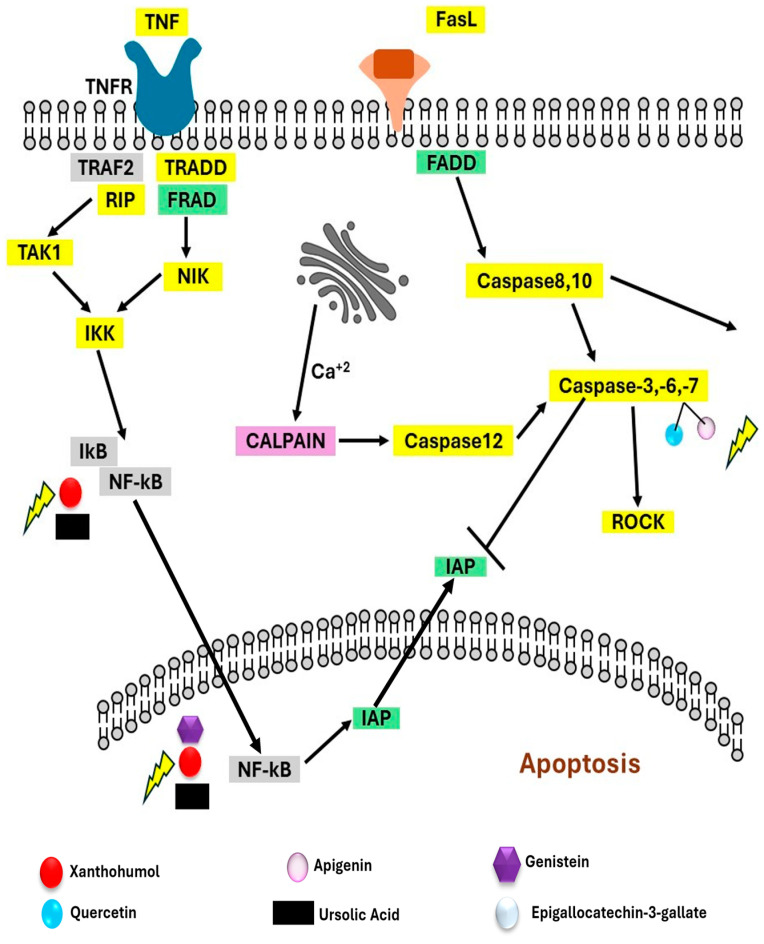
Diagrammatic representation of action mechanism on apoptosis and cell survival signalling pathways demonstrated via bioactive components of herbal medicines on breast cancer.

Herbal medicine stands as the most ancient medical system globally, with a history spanning over 2000 years [47,48]. It is a medication made entirely of plant materials such as flowers, bark, roots, seeds, leaves, and fruits. Such medicinal plants include a variety of components (polyphenols) that considerably reduce the growth of many cancers due to their powerful anti-cancerous qualities, which also help the body fight cancer more effectively and reduce the unpleasant side effects of chemotherapy and radiotherapy. Plant-derived therapeutic approaches have demonstrated their efficacy in combating diverse forms of cancer, including breast cancer, yielding positive outcomes. According to research done on ladies receiving therapy for initial-stage BC, 10.6% were taking one or more herbal treatments at the time of diagnosis, and another 28.1% began using herbal remedies following surgery. Likewise, a global survey revealed that 35.9% of individuals diagnosed with cancer had employed complementary and alternative therapies at some point, either in the past or presently. Herbal medications were by far the most often utilized group of therapies, with utilization rates witnessing an increase from 5.3% before cancer diagnosis to 13.9% following cancer diagnosis. Herbal medications are commonly utilized for two primary purposes: firstly, to alleviate the symptoms of diseases, and secondly, to serve as preventive measures against illnesses [49,50]. Several bioactive chemicals found in medicinal plants with anti-breast cancer properties have been isolated and studied for their anticancer effects on breast cancer. In general, phytochemical substances isolated from medicinal plants provide insights into their mechanisms through their activities on various signalling pathways such as apoptosis, cell cycle arrest, and autophagy. Several plant-based compounds, including quercetin and apigenin, showed apoptosis action on breast cancer by activating FASL, which mediates extrinsic apoptotic behavior, and by interacting through the TNF receptor, which mediates the activation of NF-kB transcriptional factor activities (Figure 6) [51]. Various reports were available on plant-based breast cancer therapy [52]. Garcia et al. [53] utilized mango plants for the remediation of BC. In this work, they employed Vimang, an extract of the bark of the mango plant (i.e., (*M. indica*)), which shows numerous pharmacological activities comprising anti-oxidant, anti-inflammatory, anticancer, analgesic, and immunomodulatory activities. The developed methodology demonstrates promising outcomes, as the plant extract exhibits a substantial dose-dependent inhibition of proliferation against the targeted MDA-MB-231 (triple-negative) cells. The half-maximal inhibitory concentration (IC50) was determined to be 259 µg/mL. In another study, Jaglanian et al. [54] treated triple-negative breast cancer with the rosemary plant, which successfully decreased the cancer’s progression. This study investigated the impact of rosemary extract on cell proliferation, survival/apoptosis, and the Akt and mTOR signaling pathways in TN MDA-MB-231 breast cancer cells. The rosemary extract (RE) demonstrated a dose-dependent inhibition of cell proliferation and survival in MDA-MB-231 cells. Additionally, RE suppressed the phosphorylation/activation of Akt and mTOR, while promoting the cleavage of PARP, a recognized marker of apoptosis. The findings of the developed approach suggest that rosemary exhibits potent anticancer properties, specifically against triple-negative breast cancer. Moreover, rosemary appears to modulate crucial signaling molecules involved in cell proliferation and survival. Banerjee et al. [55] used the peel and pulp of *M. indica* for the cure of BC due to its extraordinary anti-BC activities. In this work, mango, known for its effective polyphenols, displayed tumor growth suppression in breast cancer xenografts in mice. This effect was attributed to the modulation of the PI3K/AKT pathway and associated microRNAs. The study reported the dose-dependent inhibition of BT-474 cell proliferation by *M. indica* pulp extract at concentrations ranging from 2.5 to 20.0 mg GAE/L. Additionally, in nude mice carrying BT-474 xenografts, the administration of *M. indica* pulp extract at a dose of 0.8 mg GAE/day for thirty-five days resulted in a significant reduction in tumor volume. These are some reports which validate that the plant-based approach is showing effective results against breast cancer.

(b) Nanomaterial-based breast cancer therapy

**Figure 7:**
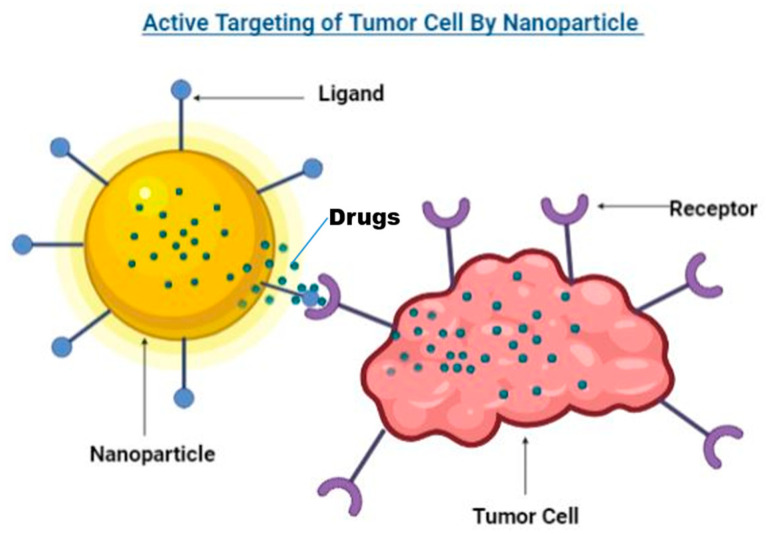
Nanoparticle-based breast cancer therapy.

Nanotechnology is the scientific field that encompasses the investigation and manipulation of materials within the scale range of one to one hundred nanometers. The concept of a “nanometer” was first introduced by Richard Zsigmondy, who was awarded the Nobel Prize in Chemistry (1925). In 1974, the term “nanotechnology” was coined by Norio Taniguchi, a distinguished professor at the University of Tokyo Science [56]. “Nanomaterials” are essential constituents of nanotechnology [57]. Physio-chemical nanoparticle properties vary significantly compared to bulk materials, primarily due to distinctions in size and morphology. Remarkably, nanoparticles acquire distinct characteristics and capabilities by undergoing alterations in their size and shape at the range of nanoscale, thereby manifesting a unique identity. Nanomaterials can be categorized according to their size and are available in various morphologies, including nanorods, nanoparticles, and nanosheets. Nanoparticles are nanomaterials characterized as zero-dimensional, while nanotubes and nanorods represent one-dimensional nanomaterials. On the other hand, two-dimensional nanomaterials consist of layer structures or films, falling into the category of type-one material. Other examples of nanomaterials include carbon, silver, gold, diamonds, zinc, zeolite, and various other substances at the nanoscale level [58,59]. Such nanomaterial has been employed in various applications for diagnosis and cancer therapy (Figure 7) [60]. Nowadays, various scientists have used nanomaterials for the remediation of breast cancer [61]. Bhagwat et al. [62] formulated solid lipid nanoparticles with transferrin targeting for breast cancer therapy. In this work, they targeted the anticancer drug tamoxifen and combined it with nanoparticles as a colloidal nano-carrier system to focus on the cells of cancer while defending normal cells. Nanoparticles were synthesized through high-pressure homogenization (HPH) utilizing lipids, surfactants, and stabilizers that are recognized as physiologically acceptable and listed as GRAS, i.e., Generally Recognized as Safe. The resulting nanoparticles exhibited an improved capability for actively targeting tamoxifen citrate in breast cancer. The formulated compounds demonstrated significantly higher cytotoxicity, in a time and concentration-dependent manner, on human breast cancer MCF-7 cells when compared to the pure solution of tamoxifen citrate. Additionally, cell uptake and flow cytometry studies established the qualitative uptake of developed D-SLN and SMD-SLN through BC MCF-7 cells. In conclusion, the work emphasizes that the utilization of transferrin-engineered nanocarriers has the potential to significantly enhance the therapeutic effect of nano-medicines in the subject of BC treatment. The application of transferrin-targeted drug delivery nanoparticles holds promise for numerous future cancer treatments. In another report, Maji R et al. [63] also synthesized tamoxifen citrate nanoparticles for the medical care of breast cancer. In this study, they repressed the nanoparticles, characterized their results via various techniques, and then combined the prepared nanoparticles with anticancer drugs. The utilization of nanoparticles as drug carriers enabled the precise delivery of medications to targeted sites, thereby facilitating the attainment of optimal drug concentrations while minimizing side effects, toxicity, dose dumping, and related concerns. No visible chemical contact was perceived between the drug and the chosen excipients in the developed methodology. The drug loadings in the prepared particles were found to be 1.5% ± 0.02% weight/weight (*w*/*w*), 2.68% ± 0.5% *w*/*w*, 4.09% ± 0.2% *w*/*w*, and 27.16% ± 2.08% *w*/*w* for NP1–NP4, respectively. Throughout the entire duration of the study, which spanned 60 days, a consistent pattern of sustained drug release was observed from the NPs. Moreover, it was seen that the MCF-7 BC cells exhibited substantial internalization of the nanoparticles in a concentration-dependent manner, with the nanoparticles predominantly localized within the cytoplasm. Notably, the nanoparticles did not enter the nucleus, indicating their limited presence within this cellular compartment. The drug-loaded nanoparticles demonstrated superior cytotoxicity compared to the free drug, implying the potential suitability of the formulation for breast cancer treatment. This efficacy is attributed to the formulation’s effective permeation into breast cancer cells, indicating its promising application in therapeutic interventions.

(c) miRNA target-based breast cancer therapy

**Figure 8:**
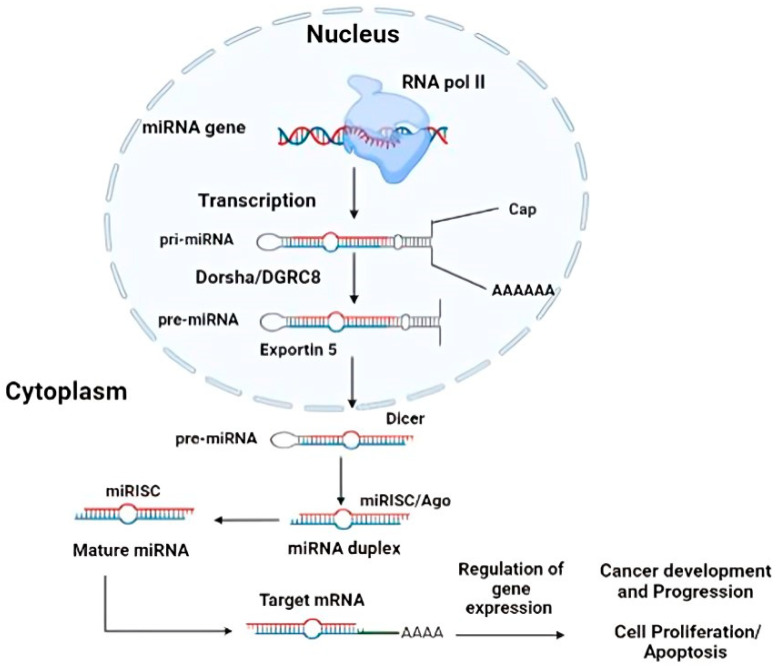
Diagrammatic representation of microRNAs biogenesis.

miRNAs are the most promising method for therapy. These are diminutive, noncoding RNA molecules that possess the ability to regulate gene expression by either activating or suppressing specific genes. These miRNAs play vital roles in the treatment of cancer, holding significant implications for cancer therapy. Hence, in the foreseeable future, the utilization of these biomolecules in the treatment of BC can be regarded as a potent tool to address the majority of common cancer-related challenges in women. Micro-RNAs (miRNAs) were initially identified in 1993 by Rhonda Feinbaum and Rosalind Lee during their research on the lin-14 gene in the nematode Caenorhabditis elegans [64]. Biogenesis of miRNAs were represented in Figure 8. The majority of miRNA genes undergo transcription within the nucleus by RNA polymerase II (Pol II) to produce primary miRNA (pri-miRNA). The processing of pri-miRNAs involves the action of a microprocessor, which consists of the double-stranded RNA-specific endoribonuclease (Drosha) and the double-stranded RNA-binding protein DiGeorge syndrome critical region 8 (DGCR8). Together, they cleave the pri-miRNAs into precursor miRNAs (pre-miRNAs). Drosha enzymatically cleaves pri-miRNA molecules, leading to the formation of hairpin-shaped precursor miRNA (pre-miRNA). Following their formation, pre-miRNAs are transported from the nucleus to the cytoplasm by exportin 5. Once in the cytoplasm, they undergo further processing by Dicer, an RNase III enzyme that interacts with both the 5′ and 3′ ends of the pre-miRNA molecules. To exert their functional effects, mature miRNAs necessitate the assembly of ribonucleoprotein complexes, such as RNA-induced silencing complexes (RISCs). Upon maturation, single-stranded mature miRNA molecules engage in interactions with Argonaute proteins (AGO) within the RNA-induced silencing complexes (RISCs). These interactions facilitate the regulation of target genes by promoting mRNA degradation or deadenylation [65]. When miRNAs are overexpressed, they can act as oncogenes by suppressing tumor-suppressor genes. Conversely, when miRNAs are underexpressed, they can function as tumor inhibitors by negatively regulating oncogenes [66].

Due to their small molecular size and their involvement in regulating tumor development and metastasis at the genomic level, miRNAs have emerged as potential therapeutic tools for silencing oncogenic miRNAs or restoring the expression levels of tumor suppressor genes. miRNAs have a relatively short half-life in circulation and face challenges in crossing hydrophobic cellular membranes, necessitating assisted delivery into cells. To overcome these limitations, miRNAs can be conjugated with high-density proteins to facilitate transportation in plasma and delivery to liver tissue. The expression of oncosuppressor miRNAs can be achieved through the transcription of short hairpin RNA (shRNA) from a plasmid vector. Additionally, the use of viral vectors expressing miRNA precursors or antagomirs represents a potential solution to address these delivery challenges [67].

### 3.2. Current Advanced Therapy for Breast Cancer 

Multiple approaches are currently emerging as promising therapeutic plans for the reliable treatment of BC, such as CRISPR-based breast cancer therapy, immunotherapy, and personalized medicine for the remediation of breast cancer.

(a) CRISPR/CAS9 technology in breast cancer therapy

CRISPR, a Nobel prize-winning technology: CRISPR is a popular strategy in the theranostic realm. In 2020, the Nobel Prize in Chemistry was awarded to CRISPR and CRISPR-related (Cas) genes, owing to their wide range of applications and significant contributions to the field [42]. Working: CRISPR is a natural adaptive immune system found in bacteria/archaea (prokaryotes) [68]. It protects bacteria from exogenous DNA, such as viruses and plasmids, which invade the cell. Such a defensive mechanism is based on short, repetitive nucleotide sequences (24–48 base pairs) surrounded by DNA fragments known as spacers. In all past infections, these PAM (protospacer adjacent motif) spacer regions were integrated into the bacterial genome. CRISPR genes are highly conserved and related to CRISPR-linked (Cas) genes. Cas protein processes CRISPR sequences to generate CRISPR RNA (crRNA), which is then used to destroy the invader organism’s DNA molecule. These molecules are complementary, permitting both RNA sequences to hybridize while inhibiting the invader’s activity. The Cas nuclease destroys this duplex, which stops the virulence mechanism and protects the cell. Furthermore, the Cas protein is in charge of adding the PAM sequence following infection to create the “memory” of the immunity. Thus, when an external agent infects the bacteria, the mechanism is activated, and the agent can be neutralized [69]. Role in breast cancer: CRISPR/Cas9 has emerged as a critical, efficient, and straightforward tool, particularly in the context of BC [70]. It provides a precise approach for the targeted insertion, correction, and removal of faulty genes associated with the disease [71]. CRISPR technology is also being used to target the oncogene HER2 exons, since a mutation in exon 12 of HER2 leads to prevalent negative mutant observable traits. The identification reveals that the mutant HER2 suppresses the MAPK/ERK axis within the HER2 signaling pathway, which is essential for the proliferation of malignant breast cells. The utilization of PARP inhibitors, known for their involvement in DNA repair and cell death processes, synergistically enhances the control of BC through CRISPR-mediated editing of HER2. This multifaceted strategy offers a potential breast cancer treatment alternative [72]. By simply redesigning an RNA sequence, CRISPR/Cas9 may target any oncogenic mutation. This technology provides an opportunity to leverage the molecular heterogeneity observed in breast cancer, allowing for personalized therapy tailored to individual patients. By doing so, it mitigates the administration of potentially harmful or ineffective medications. Individualized therapy targeting hormone receptors and HER2 receptors has significantly enhanced the prognosis of numerous individuals diagnosed with breast cancer. Nevertheless, the advent of CRISPR technology has opened up the possibility of extending these personalized therapies to individuals with triple-negative breast cancer (TNBC) [73]. Although CRISPR is extremely targeted and economically viable, its application entails the considerable risks of limiting the delivery of the altered cells again to TNBC patients, off-target effects, and combating the harmful immune response of Cas protein [74]. Various scientists utilized the CRISPR approach: Al Mulhim F et al. [75] conducted an experiment in which CRISPR/Cas9 plasmids were transfected into MCF-7 adenocarcinoma cells to either activate CDH1 or knock down CDK11. Female rats weighing between 150–190 g and aged 6–8 weeks were used for the introduction of treated cells into their mammary glands to see how well they might prevent the spread of cancer. The CDK11 gene was significantly downregulated by qPCR results, while CDH1 was significantly upregulated. Cell cycle analyses and apoptotic tests revealed that by deleting CDK11 and concurrently stimulating CDH1, the cell cycle arrest occurred specifically at the G2/M phase. Additionally, the analyses indicated a notable accumulation of cells at the G2 phase. In both genome editing hits, the proportion of cells that suffered late apoptosis rose. Histopathological sectioning data demonstrated that non-transfected MCF-7 cells possessed the capability to initiate angiogenesis and form tumors within the mammary gland. Transfected cells considerably reduced tumor localization and angiogenesis, which greatly reduced cancer cell invasion and infiltration. This study suggested that employing CRISPR/Cas9-based treatment might be a promising way to treat breast cancer, even though further research is required. In another study, Annunziato et al. [76] performed a comprehensive analysis and exploration of invasive lobular breast carcinoma (ILC), a type of breast cancer distinguished by the loss of the cell–cell adhesion protein E-cadherin. The study delved into the insights and findings regarding this particular cancer subtype. This work devised a novel methodology for the in vivo validation of potential tumor suppressors implicated in invasive lobular breast carcinoma (ILC). Female mice carrying specific alleles of the Cdh1 gene, responsible for the production of E-cadherin, can be prompted to develop invasive lobular breast carcinoma (ILC) through the administration of lentiviral vectors expressing Cre recombinase, the CRISPR/Cas9 system, or a combination of both. By employing CRISPR/Cas9 somatic gene editing, the researchers successfully directed their intervention exclusively towards ILC-initiating cells while selectively affecting the Pten gene. In mice, the development of invasive lobular breast cancer was successfully induced through the delivery of a Pten-targeting sgRNA using lentiviral vectors. This was achieved in mice with mammary gland-specific deletion of E-cadherin and expression of Cas9. Yang M et al. [77] explored the application of CRISPR/Cas9 technology to investigate the single knockout of the CXCR4 or CXCR7 gene, as well as the simultaneous knockout of both CXCR4 and CXCR7 genes, in a triple-negative breast cancer (TNBC) cell line. The individual deletion of either CXCR4 or CXCR7 genes resulted in a significant reduction in cell growth, migration, invasion, and a delay in the transition from the G1/S cycle. However, the simultaneous knockout of both CXCR4 and CXCR7 genes had a more pronounced impact on these biological functions. In both the knockout and control groups, the migration and invasion of cells treated with CXCL12 were notably higher compared to cells not exposed to CXCL12. In the group with a simultaneous knockout of CXCR4 and CXCR7, CXCL12 exhibited reduced migration and comparatively lower invasiveness compared to the single knockout groups. In summary, the knockout of the CXCR4 or CXCR7 target gene has the potential to significantly impede the progression of triple-negative BC. This intervention can effectively reduce the growth, migration, invasion, and proliferative capacity of TNBC cells. Nonetheless, the simultaneous knockout of both CXCR4 and CXCR7 proves to be highly effective. Irrespective of whether CXCR4 or CXCR7 was individually knocked out, the administration of CXCL12 enhanced the invasive and migratory capacities of triple-negative-BC. These findings present a promising target for the treatment of TNBC, highlighting the significant roles of CXCL12, CXCR4, and CXCR7 in the development of TNBC. However, further investigation is required to elucidate the precise mechanism underlying these observations.

(b) Immunotherapy-based breast cancer treatment

Meaning: Immunotherapy in cancer is described as a method of treating cancer by inducing or enhancing an immune response against it. The idea of harnessing the immune system to treat cancer has long been a topic of research [78]. Approval: The concept of relying on an immune reaction against a bacterial component to promote an anticancer immune response, established in the 1960s, led to the creation of intravesical BCG, which is currently a US Food and Drug Administration (FDA)-approved therapy for superficial bladder cancer. This achievement has been attributed to the activation of the innate immune system and an inflammatory response capable of destroying tumor cells [79]. Failures and successes: Data from the mid-1980s demonstrating the effectiveness of interleukin-2 (IL-2) in treating patients with kidney cancer or melanoma are a noteworthy example of success. This immunotherapy was extremely hazardous and did not function for other tumor types, even if it helped a tiny percentage of patients. The ability of some T cells in human tumors to identify antigens produced by those tumors, and the fact that many of these antigens were shared by several tumors, is further evidence that supports the efficacy of immunotherapy. These results raised hopes that the T and B cells that make up the adaptive immune system, each of which has a large repertoire of clonally elaborated receptors, could be trained to recognize cancer, destroy tumor cells, and create immunologic memory that would last for a long time and protect against tumor repetition [80]. Immunotherapy in Breast cancer: The administration of immunotherapeutic methods to treat cancer is presently increasingly common [81]. The initial forms of immune-based therapies for cancer centered on the utilization of humanized monoclonal antibodies capable of targeting and neutralizing specific aberrant molecules produced by cancer cells. These molecules are crucial for the existence and proliferation of cancer cells. Along with trastuzumab, various anti-HER2 monoclonal antibodies, including afatinib, gefitinib, neratinib, or lapatinib, individually or in conjunction with standard therapies, have helped to expand the range of therapeutic choices available to breast cancer patients. The anti-PD-L1 antibody atezolizumab received authorization in March 2019 for usage in combination with nab-paclitaxel to treat individuals with disseminated TNBC. This marked the inaugural approval of an immune checkpoint blockade (ICB) agent for the remediation of breast cancer. This preliminary endorsement has stoked interest in researching immunotherapy treatments for breast cancer patients [82,83]. In contrast to antibody-based immunotherapy, T cell-based immunotherapy employs cytotoxic T lymphocytes (CTLs) as effective agents for eliminating tumor cells. T cell-based immunotherapy encompasses various approaches, such as cancer vaccine immunotherapy, adoptive T cell transfer immunotherapy, and T cell receptor gene transfer immunotherapy, all aimed at harnessing the potential of T cells in combating cancer [84]. Immune checkpoint inhibitors (ICIs) have significantly changed how cancer is treated, especially for initial-phase HER2+ cancer and TNBC (triple-negative breast cancer). Leukemia inhibitory factor (LIF), a fresh immunomarker, was recently discovered. Consequently, directing therapeutic interventions towards the LIF axis may emerge as a promising approach to enhance the effectiveness of immune checkpoint inhibitor (ICI) therapy in patients with PD-L1 positive breast cancer. Although these medications have revolutionized the treatment of cancer, not all breast cancer patients, especially those with HR+ breast cancer, benefit from them. Better results may come from concentrating on the LIF axis and identifying biomarkers. Utilizing LIF as a means to enhance the efficacy of immune checkpoint inhibitor (ICI) treatment in breast cancer patients exhibiting PD-L1 positivity may be a valuable strategy [85]. Various reports have been given on immunotherapy-based breast cancer treatment. Bi Lian et al. [86] presented the advantages and drawbacks of using histone deacetylase (HDAC) inhibitors to enhance breast cancer immunotherapy. This research emphasizes that only five HDAC inhibitors—vorinostat, belinostat, panobinostat, pracinostat, and romidepsin—have principally demonstrated therapeutic benefits in hematological malignancies and have been licensed by the FDA for cancer treatment. Their effectiveness in solid tumors, including breast cancer, remains to be determined. A class of HDAC inhibitors called sirtuin inhibitors has also shown activity in several cellular processes associated with cancer. Because of their role in breast cancer immunotherapy, the development of selective and efficient histone deacetylase (HDAC) inhibitors is of utmost importance for the implementation of treatment strategies based on the distinct expression patterns of HDAC isoforms. HDAC inhibitors can also lead to negative effects such as drug resistance. The concomitant use of HDAC inhibitors, immunotherapy, and additional inhibitors in a triple combination therapy demonstrates potential in the realm of BC treatment. In an alternate investigation, Alva As and their co-workers [87] showed a study of a cohort of 28 patients between October 2016 and July 2018 with high tumor mutational burden (HTMB). HTMB mutation rates per megabase ranged from 9 to 37 in all patient tumors. The research discovered that 21% of patients (95% CI, 8 to 41) had an objective response, while 37% of patients (95% CI, 21 to 50) achieved disease control. When compared to the median overall survival, the median progression-free survival (PFS) was observed to be 10.6 weeks (95% confidence interval: 7.7 to 21.1), while the median overall survival was 30.6 weeks (18.3 to 103.3) based on the data analysis. There was no correlation seen between tumor mutational load and PFS. As stated in the product description, five patients experienced at least one significant adverse event or grade 3 adverse event that was deemed likely to be associated with pembrolizumab. Pembrolizumab monotherapy demonstrates anticancer efficacy in individuals diagnosed by heavily pretreated metastatic BC characterized through HTMB, i.e., high tumor mutational burden. The outcomes of the study align with the recent approval by the US Food and Drug Administration (FDA) of pembrolizumab as a treatment option for patients diagnosed with metastatic or unresectable solid tumors who are ineligible for alternative therapeutic approaches. Baradia A and their co-workers [88] described a study involving a cohort of 468 individuals without brain metastases. 235 patients were administered sacituzumab govitecan, while 233 patients received chemotherapy. All participants in the study had previously received taxanes and had an average age of 54. When treated with chemotherapy, the median progression-free survival was 1.7 months, whereas it increased to 5.6 months with sacituzumab govitecan. Similarly, the median overall survival with chemotherapy was 6.7 months (95% confidence interval: 5.8 to 7.7), compared to 12.1 months (95% confidence interval: 10.7 to 14.0) with sacituzumab govitecan. Furthermore, only 5% of patients exhibited an objective response with chemotherapy, whereas 35% demonstrated such a response with sacituzumab govitecan. In each group, there were three deaths as a result of adverse events, although no deaths were thought to be connected to the use of the drug sacituzumab govitecan. Patients diagnosed with metastatic TNBC who were administered sacituzumab govitecan experienced substantially increased overall survival and progression-free survival compared to those who received single-agent chemotherapy. With sacituzumab govitecan, myelosuppression and diarrhea were more prevalent. Rodriguez-Garcia A et al. [89] in their study illustrate how effective immunotherapy is severely hampered by the immunosuppressive tumor microenvironment (TME). TAMs, i.e., tumor-associated macrophages, are highly adaptable and heterogeneous cellular components of the tumor microenvironment (TME). TAMs can exhibit either M2-like characteristics, promoting tumor growth, or M1-like characteristics, enhancing antitumor immune responses. Notably, specific TAMs expressing the folate receptor (FR) demonstrate a phenotype similar to the immunosuppressive M2 subset. In mouse models of syngeneic tumors, targeted elimination of folate receptor-positive (FR+) TAMs within the tumor microenvironment (TME) using chimeric antigen receptor (CAR)-T cells leads to an increase in pro-inflammatory monocytes, recruitment of endogenous tumor-specific CD8+ T cells, a deceleration in tumor growth, and an extension of overall survival. The effectiveness of tumor-targeted anti-mesothelin chimeric antigen receptor (CAR)-T cells is enhanced by pre-conditioning the tumor microenvironment (TME) with folate receptor (FR)-specific CAR-T cells. However, simultaneous administration of both CAR products does not yield the same level of improvement in efficacy. These findings underscore the tumor-promoting role of folate receptor-positive (FR+) TAMs in the tumor microenvironment (TME) and highlight the potential therapeutic application of TAM-depleting agents as adjunctive treatments to complement conventional immunotherapies that selectively target tumor antigens.

(c) Personalized medicine-based breast cancer treatment

Precision medicine remains an important source for reducing the off-target toxicity of chemotherapeutic drugs and maximizing patient benefits [90]. This is an important technique for improving illness treatment and prevention. Precision-medicine strategies rely on the identification of appropriate biomarkers to predict the efficacy of focused therapy in a specific group of patients. Several druggable mutations have been found in breast cancer patients. Recent advances in omics technology have centered on more precise tactics for precision treatment. The advancement of next-generation sequencing technology has fuelled optimism for precision-medicine treatment options in breast cancer (BC) and triple-negative breast cancer. Potential treatment approaches for BC and TNBC include immune checkpoint inhibitors (ICIs), epidermal growth factor receptor inhibitors (EGFRi), poly (ADP-ribose) polymerase inhibitors (PARPi), antibody–drug conjugates (ADCs), oncolytic viruses (OVs), glucose transporter-1 inhibitors (GLUT1i), and signalling pathway targeting [91]. Before developing a treatment plan, modern personalized medicine considers a patient’s genetic profile and medical history. This contrasts with conventional personalized medicine, which utilizes a patient’s familial background, social circumstances, environmental factors, and lifestyle as the foundation for treatment decisions. Targeted treatment is the foundation of modern personalized medicine. The knowledge of the altered route and the elements contributing to cancer is crucial for targeted therapy. As an example, female patients diagnosed with breast cancer exhibiting elevated levels of HER-2 expression are prescribed Herceptin for treatment [92]. Trastuzumab, also known as Herceptin, is a humanized monoclonal antibody approved in 1998 for invasive breast tumor treatment characterized by the amplification of the HER-2 receptor tyrosine kinase, which occurs in nearly twenty percent of cases. Its approval was accompanied by the availability of a screening test to detect HER-2 overexpression. Herceptin has arisen as a key therapy substitute in the cases of adjuvant and metastatic instances, since it was previously established that HER-2 amplification had a negative prognostic relevance [93]. In practically all known cancer genes, the mutation is uncommon. The expanding knowledge in breast cancer genetics, facilitated by the resequencing of cancer genomes and genome-wide association studies, has covered the way for the emergence of new therapeutic approaches in BC treatment. The noteworthy illustration of BRCA1 methylation lies in its potential to function as a predictive marker in the clinical management of patients. The detection and understanding of epigenetic alterations in malignancies such as this hold great promise in expanding the scope of personalized therapy to a broader patient population. The investigation of PARP inhibitors and platinum-based drugs in clinical trials has generated significant interest in the application of DNA-damaging anticancer agents for malignancies occurring in individuals with BRCA1 or BRCA2 mutations. Notably, the inactivation of BRCA1 through epigenetic mechanisms in sporadic breast tumors elicits similar effects to those observed in genetically inherited cases with BRCA1 mutations. Hence, it is reasonable to hypothesize that these medications will exhibit comparable efficacy in cases of sporadic acquired BRCA1 methylation as they do in cases of inherited BRCA1 mutations. This notion has been validated by several investigations [94]. In situations where breast cancer has advanced to its terminal stage without receiving any treatment, surgical intervention is often recommended by physicians.

### 3.3. Robotic-Based Surgical Treatment of Breast Cancer

Thanks to its adaptability, robotic research is now a benefit to every business [95]. It can be utilized in every discipline and may even become a viable contender in the diagnostic field, as well as in illness therapies. The utilization of robotic systems offers several advantages, encompassing the reduction of labor burden, enlargement of experimental scale, acceleration of testing development processes, facilitation of larger experiments, and elimination of the requirement for additional laboratory experts to interpret results within experimental laboratories [42].

**Figure 9:**
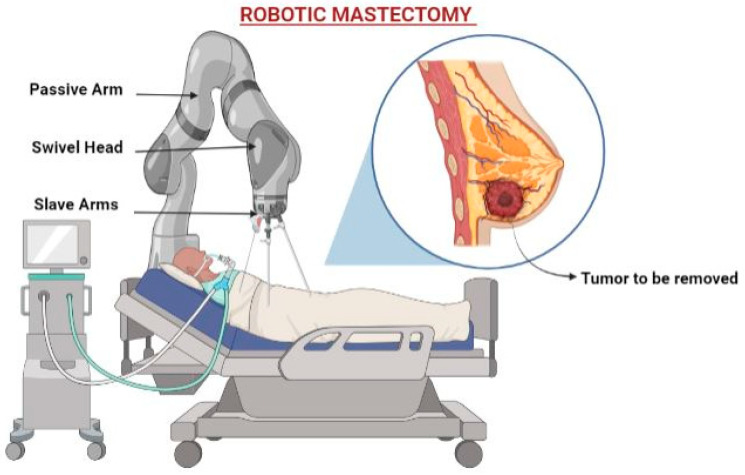
General schematic representation of robotic-based breast cancer therapy.

Over the last decade, the use of robotic technology in many disciplines has had a substantial influence on surgical approaches and patient outcomes. Robotic surgery is now seen as a viable alternative, with the bulk of cases devoted to oncologic treatments (Figure 9). In recent times, applications of robotic surgery have emerged in the field of superficial organ procedures, including thyroidectomy and oropharyngeal surgery, as well as cosmetic and reconstructive surgery, even without the natural cavity required for endoscopic imaging. Few scientists have explained the treatment of breast cancer by the robotic-based surgical approach. Toesca et al. [96] utilized robotic technology for the therapy of breast cancer. This study explore a novel technique aimed at enhancing aesthetic outcomes and the satisfaction of the patient without affecting the breast’s natural appearance and getting rid of the breast glandular tissue. By doing so, it overcomes the limitations previously encountered with the minimally invasive endoscopic approach. Therefore, the authors present the results of a consecutive series of twenty-nine procedures carried out on women with breast cancer to assess the viability, repeatability, and safety of this strategy. The developed platform demonstrated favorable outcomes, with an average total duration of approximately 3 h for the final robotic surgeries. This indicates a notably rapid learning curve associated with the procedure. No significant complications, such as hematoma, seroma, skin/nipple/areola injury, necrosis, or infection, were witnessed in any of the cases. Only two patients experienced minor blistering in the breast skin flap due to internal electrocautery, which spontaneously resolved within 7 days without requiring definite treatment. There were no signs of systemic problems. In another study, Van Mulken and their co-workers [97] directed pioneering work to investigate the use of a specialized microsurgical robotic platform for the lymphedema associated with BC treatment. This study marks the first application of robot-assisted supermicrosurgery in humans for this particular purpose. This study presents a prospective randomized pilot investigation that compares the effectiveness of robot-assisted supermicrosurgical lymphaticovenous anastomosis (LVA) with manual LVA in the treatment of breast cancer-related lymphedema. The developed platform assesses patient outcomes at 1 and 3 months following surgery, as well as the duration of the surgical process and the quality of the anastomosis. The results indicate an improvement in patient outcomes at the 3-month follow-up evaluation. Moreover, a significant reduction in the duration of the anastomosis procedure is observed in the group using robot-supported assistance, with a decrease from an average of 33 min to 16 min. In this report, the achievability of utilizing robot-supported supermicrosurgical anastomosis in LVA, i.e., lymphaticovenous anastomosis procedures. The findings demonstrate promising results, indicating a potential future for the application of reconstructive supermicrosurgery. These reports provide validation for the increasing promise of robotic-based surgical approaches in successful BC treatment, surpassing the efficacy of traditional surgical approaches.

**Table 3:** Presented various breast cancer treatments along with their advantages and disadvantages.

S. No.	Breast Cancer Treatment	Source	Advantages	Disadvantages	References
1.	Traditional Molecular Engineering-Based Therapy	Plant-based therapy	-Lowers the side effects of chemotherapy or radiation treatment. -Increases general well-being and offers immune support, which could be helpful for patients undergoing cancer management.	-Lacks the thorough clinical studies required to demonstrate safety and efficacy in treating or preventing breast cancer. -Herbal products are not as strictly controlled as pharmaceuticals; thus, the purity, dose, and quality of herbal cures can vary greatly.	[98,99]
		Nanomaterial based therapy	-Nanomaterials can improve medication delivery to cancer cells through active and passive targeting mechanisms. -Nanoparticles protect medicines against degradation, extending their half-life and ensuring their stability in the body.	-Some nanomaterials are hazardous to the liver, kidneys, and brain if not prepared and delivered appropriately. -Producing nanomaterials for cancer therapy requires careful control over particle size, surface chemistry, and drug loading. -Only a few nanomaterial-based medicines have gained extensive clinical usage for breast cancer, owing to regulatory barriers and long-term safety concerns.	[100,101]
		miRNA targeting-based therapy.	-miRNAs can selectively control gene expression, enabling the precision targeting of cancer-related pathways. For example, miR-21 and miR-155 are frequently overexpressed in breast cancer, and lowering their levels can slow tumor growth by controlling apoptosis, cell proliferation, and immunological responses. -miRNA-based treatments can make tumours resistant to chemotherapy. For example, miR-29 downregulation has been associated with chemotherapy resistance, and increasing its levels can boost sensitivity to treatment.	-miRNAs are freely destroyed in circulation, necessitating improved delivery techniques, such as nanoparticles. -Despite encouraging preclinical outcomes, miRNA-based treatment are still in the early phases of research. Their clinical translatability has yet to be thoroughly demonstrated. -Because breast cancer is so diverse, it is challenging to discover universally effective miRNA targets that work for all patients.	[102,103,104]
2.	Current Advanced Therapy	CRISPR-based therapy	-CRISPR enables precise editing of genes implicated in cancer development and metastasis. -It has the potential to enhance breast cancer therapy by knocking off oncogenes or repairing faulty tumor suppressor genes.	-One of the main issues with CRISPR is the likelihood of immunogenic toxicity, or accidental gene modifications, which might result in undesirable changes and raise the risk of new tumors.	[105,106,107]
		Immunotherapy	Provides longer-term protection, perhaps leading to remission even after therapy is discontinued, particularly in aggressive types such as triple-negative breast cancer (TNBC). -It can be used with other therapies such as chemotherapy or targeted therapy, increasing overall treatment results in certain patient populations.	Side effects include inflammation, skin rashes, and autoimmune responses, which may necessitate extra therapy. -Immunotherapy is quite expensive and may not be completely reimbursed by insurance, which limits access for certain patients.	[108,109]
		Personalized medicine-based therapy	-Treatments can be tailored to an individual’s genetic profile, increasing their efficacy. -Targeting certain genetic abnormalities, such as HER2 mutations, can greatly improve outcomes for breast cancer.	-Genetic testing and the creation of personalized treatments are costly. -While DNA sequencing prices have dropped, medicines based on genetic profile are still financially prohibitive for many patients.	[110]
3.	Surgery	Robotic-based surgical treatment of breast cancer	-Minimizes incisions compared to standard open operations. -Provides surgeons with better accuracy and control, allowing for more comprehensive cancer removal while protecting healthy tissues.	-The expenses of robotic systems, including maintenance and disposable devices, are considerably greater. -Robotic-assisted mastectomy necessitates specialized training, which takes months to master. As a result, just a few centers throughout the world now provide it.	[111,112,113]

## 4. Conclusions

The most popular form of invasive cancer, and the second leading cause of fatalities in women, is breast cancer. This is primarily due to substantial delays in diagnosis and inadequate treatment alternatives. As a result, early detection and the ability to evaluate therapy response are critical. In this overview are highlighted some of the most advanced ways of breast cancer diagnosis, such as conventional diagnostic tools and ultramodern approaches, e.g., biosensors. In addition to diagnosis, some advanced therapy and surgical options that help to lower the burden of cancer management were also illustrated. Breast cancer death rates must be reduced via effective and prompt detection and treatment of the illness. The tools for detecting and treating breast cancer have significantly improved during the last 20 years. The focus has shifted towards achieving effective control and management of the disease while minimizing patient discomfort, enhancing patient compliance, and reducing off-target side effects. Sensors are increasingly being utilized in post-operative and chemotherapy follow-ups to monitor patient health and discover issues early. Wearable sensors monitor vital indicators such as heart rate and temperature to detect illnesses or anomalies. Implantable sensors monitor internal processes, such as glucose levels, and ensure regulated medication distribution, particularly during chemotherapy. Biosensors measure biological indicators to determine therapy efficacy and avoid medication toxicity. Smart bandages with wound sensors monitor healing and help avoid post-surgical infections. Remote patient monitoring (RPM) devices send health data from home, minimizing the need for hospitalization. The adoption of 5G technology improves these capabilities through real-time data transfer, allowing for faster interventions and personalized treatment. Researchers have employed various therapies, including nanomaterials, herbal treatment, CRISPR, and immunotherapy, to improve the effectiveness of treating breast cancer. These innovative approaches have been explored to improve the outcomes and effectiveness of the therapeutic interventions associated with BC. The use of nanomedicine enables the targeted delivery and controlled release of encapsulated medications at the tumor site, modifying bioavailability and drug pharmacokinetics. Herbal-based treatment is more reliable due to its natural properties, which make the treatment more beneficial. Other than enhanced diagnosis and therapy, surgery is the most effective way to treat cancer. There are different surgical options available, but robotic-based surgery is more popular owing to its time savings, low invasiveness, and high accuracy. In the future, the persuasive invocation of the advanced therasurgynostic platform to cure breast cancer enhances the likelihood of saving female lives with appropriate therapy availability.

## Figures and Tables

**Table 1:** Advantages and Disadvantages of Various Breast Cancer Diagnostic Methods [18,19,20,21,22,23].

S. No.	Diagnostic Approaches	Advantages	Drawbacks
1.	Mammography	-Benefits include high sensitivity and specificity, low cost, and good tolerability. -Mammography has been shown to lower breast cancer mortality by 19%.	-Pain and anxiety, false alarms, and radiation hazards. -Limited sensitivity in thick breast tissue, where superimposition distortions could disguise abnormalities.
2.	Ultrasonography	-Suitable screening for young women, with non-invasive diagnostic procedures. -Detecting mammary gland inflammation.	-Limitations include the inability to detect microscopic masses and unusual tissue. -Reliance on the examining clinician, and low definition and resolution.
3.	MRI	-Monitoring of high-risk populations, such as those with a family history of cancer. -Suitable for people undergoing breast-conserving surgery.	-Not suited for all patients, including those with claustrophobia and contrast sensitivity. -Not recommended for large-scale screening or cancer staging.
4.	Biopsy	-Tissue biopsy is the primary method for detecting and diagnosing tumors. -It involves analyzing tissue at the cellular level to identify aberrant or malignant cells.	-The limitations of this method include restricted tissue sample availability. -Low sensitivity and accuracy., high procedure costs. -Difficulty distinguishing between tumor types and invasiveness.
Smart diagnostic tool		Advantages	
5.	Biosensor	-Provides quick, on-site detection, easy and cost-effective approaches. -Because of their low abundance, glycoproteins are the recommended targets for breast cancer diagnostics above miRs and CTC. -Compared to antibodies, aptamers have the advantage of being synthetic and thermally stable, making them ideal candidates for bio-detection. -Enables the sensitive and selective detection of BC.	

## Data Availability

Data will be available on request.
